# Identification and characteristics of multiple viruses in snow leopard fecal samples using viral metagenomics

**DOI:** 10.3389/fmicb.2026.1946718

**Published:** 2026-09-30

**Authors:** Yue Xue, Shiyu Cai, Li Ji, Likai Ji, Quan Shen, Xiaochun Wang, Ping Wu, Yuwei Liu, Yan Wang, Wen Zhang, Shixing Yang

**Affiliations:** 1 School of Medicine, Jiangsu University, Zhenjiang, Jiangsu, China; 2 The Affiliated Zhenjiang Mental Health Center, Jiangsu University, Zhenjiang, Jiangsu, China

**Keywords:** gut virome, phylogenetic analysis, sequence alignment, snow leopard, viral metagenomics

## Abstract

Metagenomic studies on the fecal virome of snow leopards, rare apex predators on the Qinghai-Tibet Plateau (QTP), remain limited. In this study, we utilized metagenomic sequencing to characterize the fecal viral community in captive QTP snow leopards. We assembled 22 complete viral genomes across the families Anelloviridae, Circoviridae, Parvoviridae, Picornaviridae, Genomoviridae, and Smacoviridae. Phylogenetic analyses indicated substantial sequence novelty. We identified 9 anellovirus genomes in the genera *Tettorquevirus* and *Etatorquevirus*, 3 candidate novel parvoviruses in *Bocaparvovirus*, *Chaphamaparvovirus*, and *Dependoparvovirus*, 3 circovirus genomes representing 2 candidate novel species, and 1 candidate novel sakobuvirus species. Another kobuvirus genome represented a divergent strain of a known species. In addition, 4 divergent genomoviruses showed phylogenetic relationships with sequences reported from diverse animal and environmental sources. Notably, one candidate novel porprismacovirus (family Smacoviridae) displayed a marked discordance between its highly conserved replication-associated protein (96.7% amino acid identity), divergent capsid protein (maximum identity, 59.7%), and low whole-genome identity (63.6%). Recombination analysis suggested it might be a recombinant virus. In summary, this study expands current knowledge of the diversity and evolutionary characteristics of viruses detected in snow leopard fecal samples and provides baseline genomic data for future investigation of captive snow leopard viromes.

## Introduction

The gut virome of wildlife is widely considered an important source of viral diversity and a potential reservoir for emerging and re-emerging viruses (Esposito et al., 2022). In the context of global ecological changes and continuously expanding human activities, human-animal interfaces have become increasingly frequent (Ellwanger and Chies, 2021). Such interactions across ecological boundaries may increase opportunities for cross-species spillover (Bhatia et al., 2024) and spillback (Fagre et al., 2022) of zoonotic pathogens. Therefore, monitoring the diversity of viruses in wildlife populations at specific contact interfaces is relevant to the prevention and control of potential emerging infectious diseases (French and Holmes, 2020). In recent years, metagenomic sequencing, with its advantage of not relying on prior sequence information, has emerged as a core tool for characterizing complex host viromes and uncovering previously undescribed viruses (Zhang et al., 2018). The snow leopard (Panthera uncia), a rare apex predator in alpine mountain ecosystems such as the Qinghai-Tibet Plateau, is currently listed as a Vulnerable species by the International Union for Conservation of Nature (IUCN) (McCarthy et al., 2017). Due to the elusive nature of snow leopards and the inaccessibility of their natural habitats, epidemiological data on infectious diseases in free-ranging snow leopard populations remain limited (Esson et al., 2019). However, with the improvement of wildlife rescue mechanisms, some wild snow leopards that have lost their ability to survive in the wild have been accommodated in wildlife parks (e.g., the Qinghai Wildlife Park) for *ex situ* conservation. This special population may retain baseline microbiological traits associated with the natural wild environment while also being subjected to prolonged exposure to captive conditions (McKenzie et al., 2017). During routine zoo husbandry and management, interactions between snow leopards and personnel, such as zookeepers and veterinarians, constitute a typical human-animal interface. Furthermore, the relatively restricted living space, specific artificial diets, and potential spatial overlap with other captive species may increase opportunities for exposure to viruses associated with other animals, feed, personnel, or the environment and thus provide a useful setting in which these relationships can be investigated (McAloose et al., 2020; Di Profio et al., 2025). Limited studies on the gut microecology of carnivores indicate that their viral community composition can be highly dynamic. Under conditions of captivity and artificial intervention, common enteric viruses, such as those from the families Parvoviridae and Picornaviridae (e.g., *Kobuvirus*), may be detected in captive animals, and their occurrence may be influenced by environmental exposure within shared spaces (Di Martino et al., 2021). In addition, the specific dietary and environmental exposures within zoos may influence the host’s fecal virome profile. For instance, communities of circular Rep-encoding single-stranded DNA (CRESS DNA) viruses–represented by the families Anelloviridae, Circoviridae, and Smacoviridae–display substantial genetic diversity and have been detected in herbivores and environmental materials. Their detection in captive snow leopard feces may reflect host association, dietary passage, or environmental exposure. Indeed, recent molecular and metagenomic studies have begun to reveal the diversity of snow leopard viral communities. Johansson et al. (2020) reported rotavirus, felid herpesvirus 1, feline papillomavirus 2, CRESS DNA viruses, and feline anellovirus in samples from seven free-ranging snow leopards in Mongolia. A subsequent metagenomic analysis of a wild snow leopard fecal sample identified viruses with Parvoviridae, Genomoviridae, Smacoviridae, Circoviridae, Picobirnaviridae, Anelloviridae, and Adenoviridae, together with a candidate novel bocaparvovirus tentatively named Panthera uncia bocaparvovirus (PuBOV) (Chukwudozie et al., 2021). Together, these studies provide useful qualitative context for the present captive dataset and a basis for future standardized comparisons of captive and free-ranging snow leopard viromes. Although traditional viral detection methods, such as PCR and virus isolation, offer advantages in specificity, they may have limitations in breadth and sensitivity because they generally require predefined targets or cultivable viruses. The emergence of next-generation sequencing (NGS) technologies has provided a complementary technical approach for viromic studies (Satam et al., 2023). This method does not rely on predefined detection targets and can capture broad genetic information from a sample, enabling not only the detection of known viruses but also the discovery of previously undescribed or divergent strains. Furthermore, it can be combined with phylogenetic analysis to examine the evolutionary relationships of viruses (Liu et al., 2021).

This study mainly utilizes high-throughput metagenomic sequencing to characterize the fecal virome of captive snow leopards at the Qinghai Wildlife Park, with a primary focus on divergent viral genomes and a putative recombinant genome. By describing the phylogenetic relationships and sequence characteristics of viruses from the families Anelloviridae, Circoviridae, Parvoviridae, Picornaviridae, Genomoviridae, and Smacoviridae, this study expands the available genomic information for viruses detected in endangered felid feces and provides baseline genomic data for future investigation of captive snow leopard viromes.

## Materials and methods

### Sample collection and preparation

Seven freshly fecal samples from captive snow leopards were collected from the Qinghai Wildlife Park in February 2025 (three sample libraries: YFe17-YFe19) and July 2025 (four sample libraries: XY-Feces-I1-XY-Feces-I4). Within each sampling period, each sample originated from an independent snow leopard, while some animals were sampled at both time points; the sampling design was therefore descriptive rather than a strictly paired longitudinal comparison. No obvious clinical signs were recorded at sampling, and none of the sampled animals was receiving medication. Sex, age, and feed type were not recorded and were not included as covariates. Samples were collected non-invasively from enclosure surfaces during routine husbandry, without handling or contacting the animals, and with permission from the wildlife park. The sample collection process followed strict aseptic procedures. To reduce viral nucleic acid degradation, samples were immediately transported to the laboratory under cold-chain conditions. 100-mg aliquot of each sample was resuspended in 0.5 mL of Dulbecco’s phosphate-buffered saline (DPBS), vigorously vortexed for 5 min, subjected to three consecutive freeze-thaw cycles, and then centrifuged at 15,000 × *g* for 10 min at 4 °C. The resulting supernatant was transferred into a 1.5-mL centrifuge tube and stored at −80 °C for subsequent nucleic acid extraction (Chen et al., 2026).

### Library construction

The resulting supernatant (approximately 500 μL) was passed through a 0.45-μm filter (Millipore) to reduce eukaryotic cells, bacteria, and other non-viral particles (Qian et al., 2024). Subsequently, unprotected nucleic acids were digested using a cocktail of DNase and RNase at 37 °C for 60 min (Xi et al., 2023). Total nucleic acids (DNA and RNA) were extracted utilizing the QIAamp Viral RNA Mini Kit (QIAGEN) in accordance with the manufacturer’s instructions. To convert the nucleic acids—comprising both DNA and RNA viral sequences–into complementary DNA (cDNA), reverse transcription was performed using SuperScript IV (Invitrogen) along with 100 pmol of random hexamer primers. Second-strand DNA synthesis was then conducted using Klenow Fragment polymerase (New England BioLabs) (Xi et al., 2023). Finally, seven dual-indexed libraries were constructed using the Nextera XT DNA Sample Preparation Kit (Illumina) and sequenced on the Illumina NovaSeq 6000 platform using 150-bp paired-end reads (Lu et al., 2024). A double-distilled-water blank extraction control was processed in parallel through nucleic acid extraction, library preparation, sequencing, and bioinformatic screening.

### Bioinformatics analysis

The 150-bp paired-end reads generated from sequencing were processed using Illumina’s proprietary software. Primer and adapter sequences and low-quality reads were removed using Trim Galore v0.6.7 (Krueger et al., 2023) with the parameters –phred33, –length 100, –stringency 3, and –paired, retaining only high-quality sequence reads. Subsequently, the filtered reads were *de novo* assembled using MEGAHIT v1.2.9 (Li et al., 2016) with the parameter –min-contig-len 200 to generate contigs. Both the assembled contigs and the filtered sequence reads were then aligned against the NCBI viral protein database using DIAMOND v2.1.12 (Buchfink et al., 2021). To further reduce potential false-positive viral assignments, candidate viral sequences were realigned against an in-house non-redundant non-viral protein (NVNR) database downloaded in March 2025. In addition to the default parameters, the alignment was performed with an E-value threshold of 1 × 10-5 and with the options -k 25 and -f 100, which retained a maximum of 25 candidate matches per query and output the results in DAA format. These DAA files were subsequently imported into MEGAN v7.1.1 (Huson et al., 2016) and converted into RMA format for viral community analysis.

### Viral composition analysis

The taxonomic and functional abundance matrices for each library (*n* = 7) were parsed and exported using MEGAN v7.1.1. Subsequently, downstream data processing and visualization were conducted in the R v4.5.1 environment. First, the abundance data were cleaned and reshaped utilizing the dplyr and tidyr packages. Following this, stacked bar charts and pie charts were generated using the ggplot2 and scales packages (Wickham, 2016). These visualizations were used to display the compositional structure of the viral communities in each sample and the abundance distribution and relative proportions of the dominant taxa. Because the dataset comprised four July libraries and three February libraries, with possible repeated sampling of some animals, comparisons between sampling periods were treated as descriptive rather than as evidence of seasonal virome patterns.

### Viral sequence extension, annotation, and phylogenetic analysis

Contigs exhibiting significant similarity to known viruses based on BLASTx results were selected for further investigation. Contigs exceeding 500 bp in length were subjected to downstream analysis. Using each contig as a reference, the raw reads from their corresponding original barcodes were mapped utilizing the Low Sensitivity/Fastest parameters in Geneious Prime v2024.0.5 to inspect coverage, paired-read agreement, and assembly continuity. Also, within Geneious Prime v2024.0.5, specific open reading frames (ORFs) were predicted from complete or near-complete contigs that displayed similarity to known viruses. ORFs were defined by a minimum size of 400 bp and an ATG start codon. These predicted ORFs were subsequently verified by aligning them against viral proteins in the GenBank database. ORF annotation was performed utilizing the Conserved Domain Database (CDD v3.21)^1^ (Marchler-Bauer et al., 2017; Lu et al., 2020; Wang et al., 2023).

Phylogenetic analyses were conducted based on the predicted protein sequences of the viruses identified in this study, the best-matched sequences obtained from the GenBank database via BLASTx alignment (E-value cutoff < 1 × 10-5), and representative viral sequences from related viral genera or species. MUSCLE alignments of relevant protein and nucleotide sequences were performed using MEGA v11 (Tamura et al., 2021) with default settings. The resulting alignments were then trimmed to reduce phylogenetic noise using TrimAl v1.5.0 (Capella-Gutiérrez et al., 2009) with the parameter -automated1. Subsequently, maximum-likelihood phylogenetic trees were constructed using IQ-TREE 2 v2.3.6 (Minh et al., 2020). The best-fitting substitution model for each alignment was selected by ModelFinder according to the Bayesian information criterion (Kalyaanamoorthy et al., 2017), and branch support for the principal virus trees was evaluated using 1,000 standard nonparametric bootstrap replicates. Finally, the phylogenetic trees were visualized and polished using ChiPlot (Xie et al., 2023)^2^ and Adobe Illustrator 2025 v29.0.

### Recombination and assembly-continuity analyses

To test whether Sum-sma-SnowLeopard-1 is a recombinant, the complete genome sequences of eight representative Porprismacovirus genomes available in the GenBank database were downloaded, then aligned with Sum-sma-SnowLeopard-1 to generate a nucleotide alignment of 3,359 positions. The resulting nucleotide alignment was screened using Recombination Detection Program 4 v4.101 (Martin et al., 2015) with nine methods: RDP (Martin and Rybicki, 2000), GENECONV (Padidam et al., 1999), BootScan (Martin et al., 2005), MaxChi (Smith, 1992), Chimaera (Posada and Crandall, 2001), SiScan (Gibbs et al., 2000), PhylPro (Weiller, 1998), LARD (Holmes et al., 1999), and 3Seq (Boni et al., 2007). A recombination signal was considered well supported when at least three methods returned corrected *P* < 0.05. The inferred event was further examined using SimPlot BootScan with a 200-bp window, a 20-bp step, gap stripping, 100 replicates, the Kimura two-parameter model, a transition/transversion ratio of 2.0, and neighbor-joining trees (Lole et al., 1999). The alignment was divided at the inferred breakpoints into a putative recombinant region (alignment positions 1,598–3,018; 1,421 nucleotides after removal of all-gap columns) and a circular background region (positions 3,019–3,359 plus 1–1,597; 1,938 nucleotides). Maximum-likelihood trees were inferred for the two regions in IQ-TREE 2 v2.3.6 with ModelFinder and 1,000 UFBoot2 replicates with NNI optimization (-bb 1000 -bnni) (Hoang et al., 2018). The selected models were TPM2u+F+I+G4 and K3Pu+F+I+G4, respectively.

### Quality control

All experimental procedures were conducted in accordance with standard laboratory precautions. Nucleic acid samples were resuspended in DEPC-treated water supplemented with RNase inhibitors. Double-distilled water (ddH2O) [Sangon Biotech (Shanghai) Co., Ltd.,] was prepared under identical conditions as a blank control for subsequent processing. The blank control was carried in parallel through nucleic acid extraction, library preparation, sequencing, and bioinformatic screening. No reads or assembled contigs assigned to the target viral genomes reported in this study were detected in the blank control. Furthermore, all pipette tips used throughout the experiments were equipped with aerosol filters. All experimental consumables that came into direct contact with the nucleic acid samples, including 1.5-mL microcentrifuge tubes and pipette tips, were certified RNase- and DNase-free. These measures were systematically implemented to prevent cross-contamination among samples and to avoid nucleic acid degradation.

## Results

### Overall view of the virome

Illumina sequencing generated 68,559,460 raw sequence reads across the seven libraries. Following bioinformatic analysis, 3,751,833 sequence reads were assigned to viral taxa, accounting for 5.47% of the total raw reads.

As illustrated in the pie chart (Figure 1A), Anelloviridae was the most abundant family among reads assigned to the six focal viral families, representing 56.8% of their total relative abundance, followed by Circoviridae (14.9%). Other major viral families included Genomoviridae (8.6%), Parvoviridae (7.5%), Smacoviridae (7.4%), and Picornaviridae (4.8%).

**FIGURE 1:**
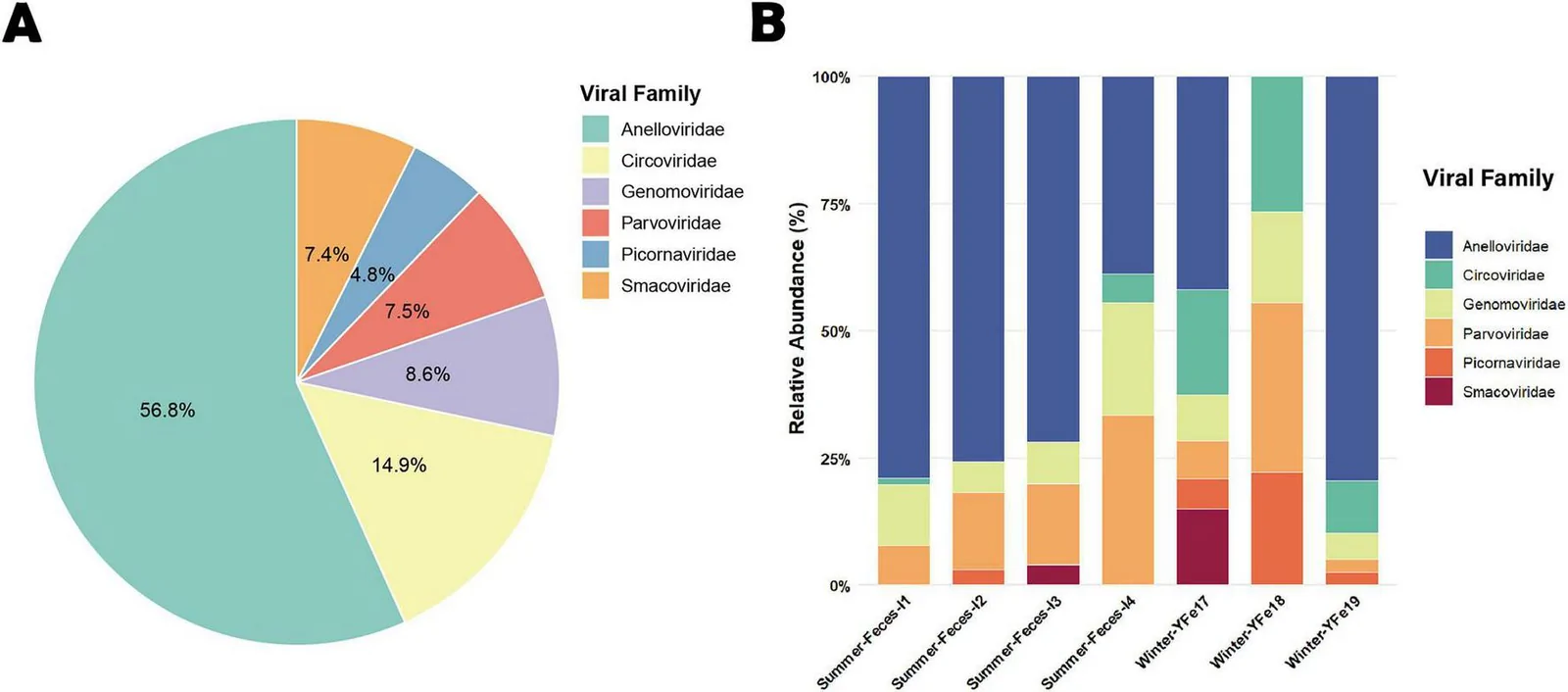
Taxonomic composition and relative abundance of the snow leopard fecal virome at the family level. **(A)** Taxonomic composition and overall relative abundance of the six focal viral families. **(B)** Relative percentage abundance of viral families across individual snow leopard fecal libraries. The values describe inter-library patterns across the two sampling periods.

To further analyze differences across individual libraries (July samples XY-Feces-I1 to I4 and February samples YFe17 to YFe19), a 100% stacked bar chart of relative abundance was constructed (Figure 1B). The results showed relative inter-library variation in viral community. In XY-Feces-I1, I2, I3, and YFe19, *anellovirius* remained at a high abundance. Parvovirus showed a higher relative contribution in XY-Feces-I4 and YFe18, whereas circovirus and *genomovirus* showed higher relative contributions in YFe17. Detailed genomic characteristics, including sequence lengths, GC content, and ORF annotations, are summarized in Supplementary Table 1.

### Identification of divergent circovirus genomes exhibiting distinct phylogenetic clustering

In this study, we assembled three circovirus genomes from the libraries and constructed a phylogenetic tree based on the Rep protein (Figure 2A). As illustrated, all three circoviruses identified from the fecal metagenomes clustered within the genus *Circovirus*; however, their phylogenetic distribution differed between sequences detected at the two sampling times. Specifically, the two summer sample sequences (Sum-cir-Snow Leopard-1 and Sum-cir-Snow Leopard-2) clustered tightly on the phylogenetic tree and formed an independent sub-clade with multiple avian-derived (especially pigeon-derived, e.g., GenBank no. PV454396, JX901128, and NC_002361) circoviruses. In stark contrast, the winter circovirus (Win-Cir-Snow Leopard-1) displayed a markedly distinct evolutionary affiliation. Rather than clustering with avian viruses, this virus shared high homology with other circoviruses derived from carnivorous mammals, including lynx (*Lynx pardinus, Lynx rufu*s) and coyotes (*Canis latrans*), forming a monophyletic clade with robust statistical confidence (100% bootstrap value).

**FIGURE 2:**
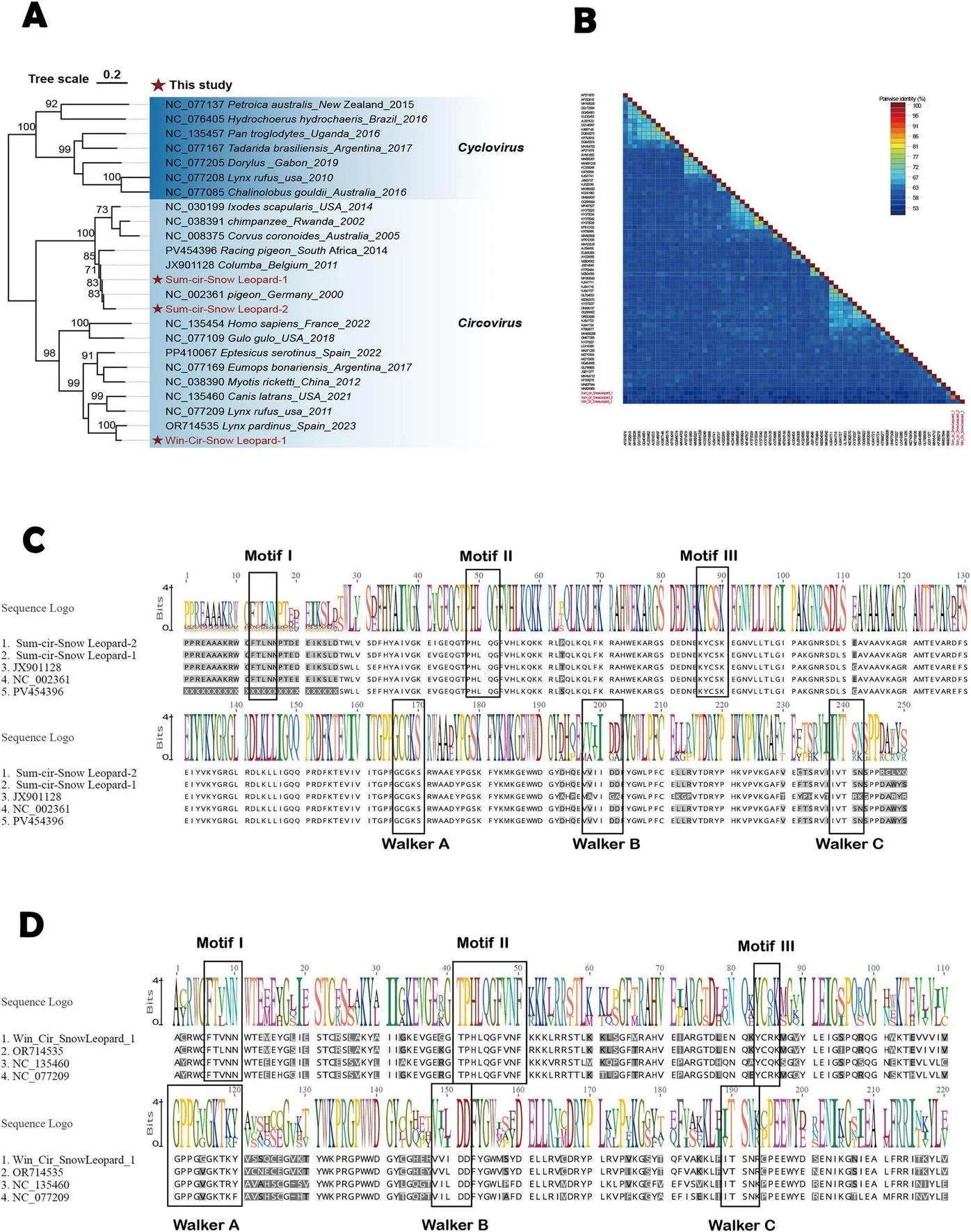
Phylogenetic and genomic characterization of circoviruses identified in snow leopard feces. **(A)** Maximum-likelihood phylogenetic tree constructed from Rep amino acid sequences using IQ-TREE 2. Node values represent support from 1,000 standard bootstrap replicates, and genomes identified in this study are highlighted with red stars. **(B)** Heatmap showing genome-wide pairwise nucleotide identity among the three circovirus genomes and representative circovirus strains. **(C,D)** Sequence logos and Rep amino acid alignments showing conserved rolling-circle replication motifs I-III and helicase motifs Walker A–C.

To determine the taxonomic status of the circoviruses identified in this study, we performed a genome-wide pairwise identity analysis between the newly identified circocviruses and corresponding known circoviruses (Figure 2B). The results revealed that the two summer circoviruses (Sum-cir-Snow Leopard-1 and Sum-cir-Snow Leopard-2) shared 88.3% nucleotide identity with each other; however, their genome-wide nucleotide identity with all other representative strains in public databases (including the phylogenetically proximate avian-associated circoviruses) was low ( < 64.5%). For the winter circovirus (Win-Cir-Snow Leopard-1), whole-genome alignment showed that its highest identity with phylogenetically related circovirus strains from carnivorous mammals (e.g., lynx) and other reference strains was 60.1%. Furthermore, its identity with the two summer circoviruses mentioned above was also approximately 60.0%. The International Committee on Taxonomy of Viruses (ICTV) criteria for species demarcation within the genus *Circovirus* require a genome-wide nucleotide similarity threshold of ≤ 80% and consistent topological localization of the origin of replication (ori) (Rosario et al., 2017). Based on these criteria, since the identities of all snow leopard-derived circoviruses identified in this study with known viruses fall far below the 80% species demarcation threshold, the two July circoviruses and the February circovirus are consistent with two candidate novel species within the genus *Circovirus*.

Since the replicase protein (Rep) of circoviruses initiates rolling-circle replication (RCR), the structural integrity of its core domains is essential for assessing whether newly discovered viral sequences retain replication potential. We performed a multiple sequence alignment of the Rep proteins from Win-Cir-Snow Leopard-1 and its closely related mammalian circoviruses within the same clade, including lynx-derived strains (GenBank no. OR714535 and NC_077209) and coyote-derived NC_135460. As illustrated in Figures 2C,D and the newly identified snow leopard-derived circovirus retains all classical RCR core motifs at the N-terminus of its Rep protein, including Motif I (FTVNN), Motif II (PHLQG), and Motif III (YCSK) (Ilyina and Koonin, 1992). Moreover, within the superfamily 3 (SF3) helicase domain, critical motifs involved in ATP binding and hydrolysis are also highly conserved, encompassing Walker A (GPPGCGKT), Walker B (V/IDD), and Walker C (ITSN). This conservation across amino acid sequences and catalytic domains supports the accuracy of the annotation and the intact coding capacity of the assembled genome, and indicates that the Rep protein retains the core molecular features associated with rolling-circle replication.

### Genomic and phylogenetic characterization of highly divergent anelloviridae genomes

Nine *anellovirus* genomes were obtained from the libraries, and a phylogenetic tree was constructed based on ORF1 protein sequences (Figure 3A). Phylogenetic analysis revealed that the nine identified anelloviruses partitioned into two distinct genus-level clades, both of which exhibited close genetic relationships with feline-derived anelloviruses. Specifically, a single July *anellovirus* (Summer-Anello-Snow leopard-1) fell within the genus *Tettorquevirus* clade and clustered tightly with a reference sequence from a domestic cat in China (GenBank no. NC_076153). The remaining eight anelloviruses, comprising five July and three February sequences, co-clustered within the genus *Etatorquevirus* clade. Within this clade, these snow leopard-derived anelloviruses formed a relatively independent and robust subclade, with their closest phylogenetic relative being an *anellovirus* from an ocelot (GenBank no. OL449682), which formed a sister group to the downstream cluster of classical domestic cat and wildcat anelloviruses. The July and February snow leopard anelloiviruses displayed an intermingled topology within the genus *Etatorquevirus* clade (e.g., Summer-Anello-Snow leopard-4 and Winter-Anello-Snow leopard-3 clustered within the same micro-clade with 94% bootstrap support).

**FIGURE 3:**
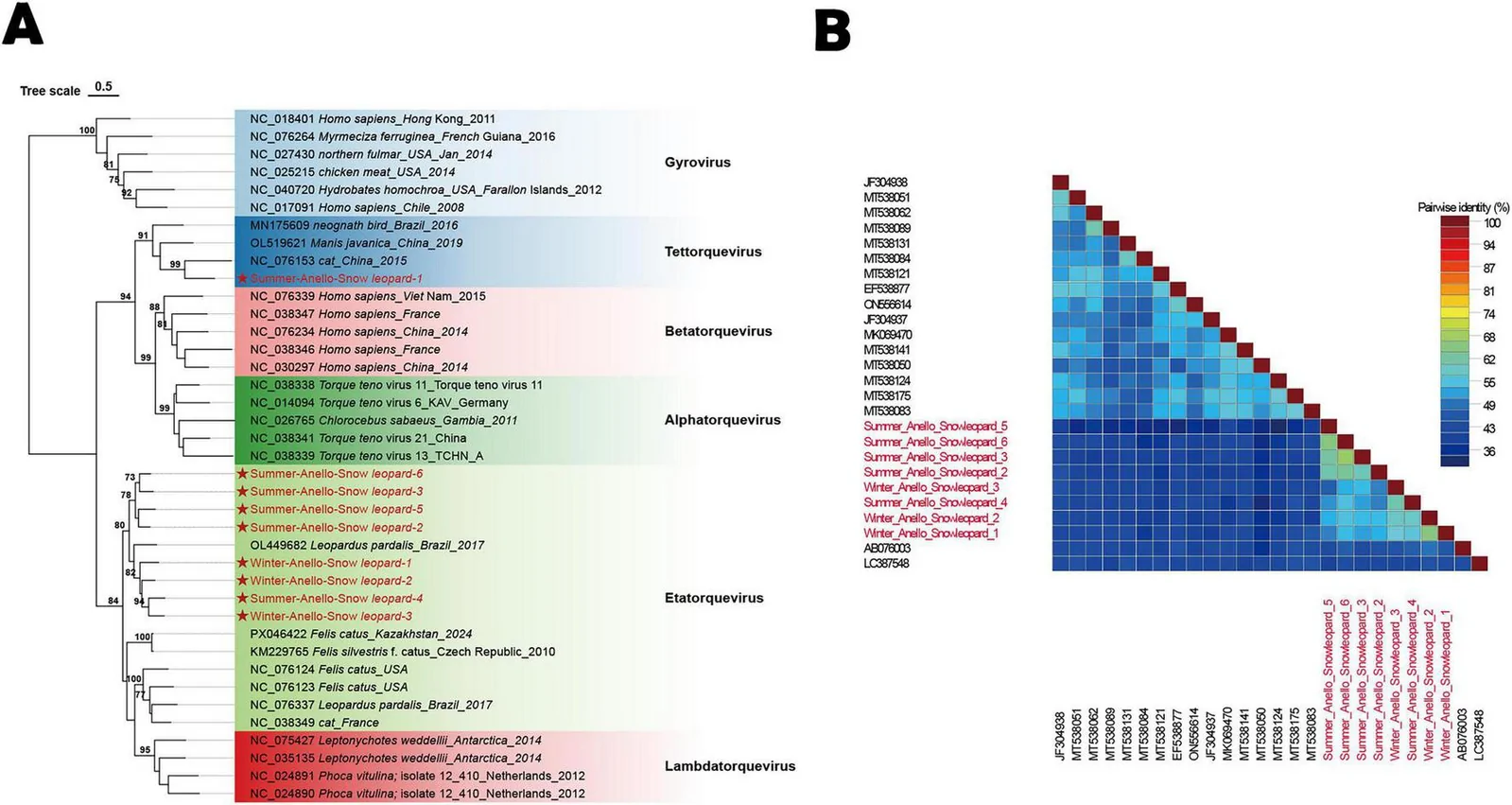
Phylogenetic and genomic characterization of anelloviruses identified in snow leopard feces. **(A)** Maximum-likelihood phylogenetic tree constructed from ORF1 amino acid sequences using IQ-TREE 2. Node values represent support from 1,000 standard bootstrap replicates. The nine divergent genomes identified in this study are highlighted with red stars and fall within the *Tettorquevirus* and *Etatorquevirus* clades. **(B)** Heatmap showing genome-wide pairwise nucleotide identity among the nine *anellovirus* genomes and representative sequences from related genera.

To further analyze the sequence divergence, we first conducted a pairwise identity analysis on the single *anellovirus* of the *Tettorquevirus* clade (Summer-Anello-Snow leopard-1) by comparing its complete genome against the known representative reference strain of this genus (GenBank no. KX262893) and several homologous sequences from public databases. Alignment results indicated that this snow leopard-derived *anellovirus* shared 30.39% nucleotide identity with the validated reference strain KX262893; even when compared with the most closely related sequence currently available in the database, the identity remained limited, peaking at only 39.07%. Next, for the eight snow leopard-derived anelloviruses belonging to the *Etatorquevirus* clade, a detailed pairwise identity matrix was constructed (Figure 3B). As shown in Figure 3B, the matrix exhibited a broad distribution pattern. The eight snow leopard-derived anelloviruses formed several subgroups with moderate to high intra-group identities ranging from 47.1 to 67.4%, among which Summer-Anello-Snow leopard-3 and Summer-Anello-Snow leopard-6 shared the highest identity (67.4%). However, when these eight sequences were compared pairwise with all other known representative *etatorquevirus* strains in public databases, the matrix displayed a large expanse of dark-blue low-value regions, with the highest nucleotide identity to any known reference sequence reaching only 45.8% (between Winter-Anello-Snow leopard-1 and a reference strain).

According to the species demarcation criteria established by the International Committee on Taxonomy of Viruses (ICTV) for the family *Anelloviridae*, nucleotide sequence divergence between different species typically exceeds 35% (i.e., < 65% identity), whereas divergence between different genera generally exceeds 44% (i.e., < 56% identity) (Varsani et al., 2021). Based on these alignment results, whether for the single anellovirus in the *Tettorquevirus* clade or the eight *anellovirues* in the *Etatorquevirus* clade, their highest identities to the included known anelloviruses were generally below the cited species and genus demarcation thresholds. These results support the presence of multiple candidate novel anellovirus taxa.

### Discovery of divergent parvovirus genomes representing three candidate novel species

In this study, we assembled three parvovirus genomes from the libraries and constructed a phylogenetic tree based on the amino acid sequences of the non-structural protein 1 (NS1) (Figure 4). As illustrated, the phylogeny was distinctly demarcated into three major subfamilies of Parvoviridae: Densovirinae, Hamaparvovirinae, and Parvovirinae. Phylogenetic analysis revealed that the three highly divergent parvovirus genomes identified in the snow leopard feces were scattered across different subfamilies and evolutionary clades, indicating broad phylogenetic diversity.

**FIGURE 4:**
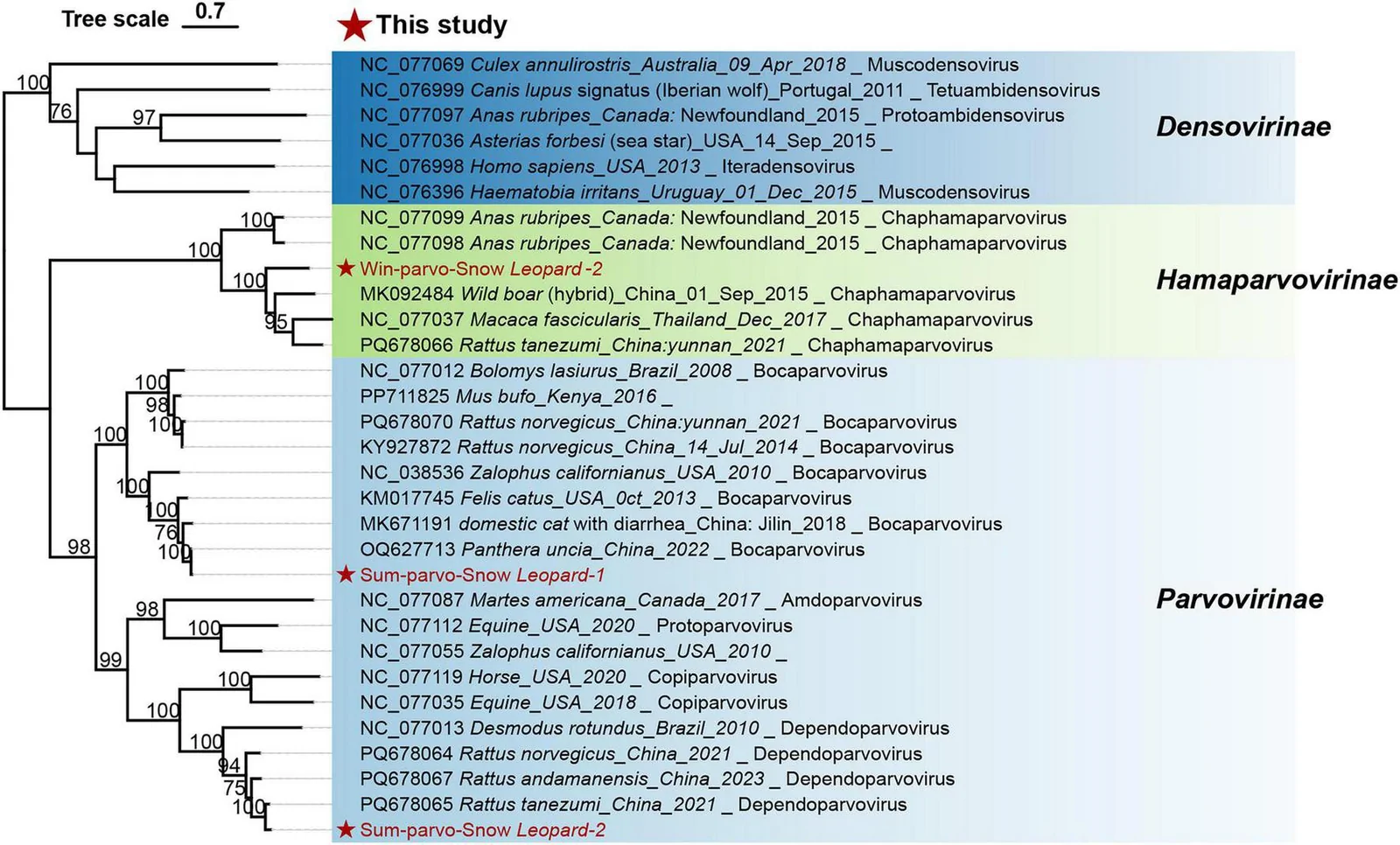
Phylogeny of parvoviruses identified in snow leopard feces. The maximum-likelihood tree was constructed from NS1 amino acid sequences using IQ-TREE 2. Node values represent support from 1,000 standard bootstrap replicates. The three candidate novel parvovirus genomes identified in this study are highlighted with red stars and fall within the subfamilies Hamaparvovirinae and Parvovirinae.

First, the winter snow leopard parvovirus (Win-parvo-Snow Leopard-2) was placed within the genus *Chaphamaparvovirus* within the subfamily Hamaparvovirinae. The topology indicated that this virus formed a robust monophyletic group (bootstrap support = 100%) with multiple reference viruses derived from non-feline hosts (e.g., wild boar GenBank no. MK092484, cynomolgus macaque *Macaca fascicularis* GenBank no. NC_077037, and Asian house rat *Rattus tanezumi* GenBank no. PQ678066), sharing the closest phylogenetic relationship with the wild boar and rat-derived strains (bootstrap support = 95%). Because this clade contains viruses reported from ungulate and rodent hosts, dietary passage represents a plausible interpretation supported by the phylogenetic relationship and a focused hypothesis for assessment using paired dietary and environmental samples.

Second, the two parvoviruses identified in the summer samples exhibited characteristics of host association and sequence divergence. Specifically, Sum-parvo-Snow Leopard-1 was assigned to the genus *Bocaparvovirus* within the subfamily Parvovirinae. Within the *Bocaparvovirus* clade, this virus clustered with a reference parvovirus from a snow leopard published in 2022 (GenBank no. OQ627713, *Panthera uncia*), and together with bocaviruses derived from domestic cats (GenBank no. MK671191 and KM017745), formed a supported felid-associated clade (basal bootstrap support = 100%). The supported clustering of Sum-parvo-Snow Leopard-1 with sequences reported from the same host species and other felids is consistent with a felid-associated lineage and supports targeted investigation of its persistence and host association in snow leopards.

Finally, although the other summer parvovirus (Sum-parvo-Snow Leopard-2) was also located within the subfamily Parvovirinae, it positioned at the most basal branch of the genus *Dependoparvovirus*. Within the broad *Dependoparvovirus* clade (basal bootstrap support = 99%), Sum-parvo-Snow Leopard-2 formed a highly divergent, independent evolutionary lineage, situated outside branches encompassing *dependoparvoviruses* associated with diverse mammals such as rats, bats, and horses. Such a highly outgrouped topological position and significant sequence divergence suggest that Sum-parvo-Snow Leopard-2 may represent a candidate novel viral species within this genus.

To further evaluate the candidate species status of the three parvoviruses identified in this study, we utilized the Sequence Demarcation Tool (SDT) (Muhire et al., 2014) to calculate their pairwise identity matrices against relevant reference strains based on the amino acid sequences of the NS1 protein. The selection of comparison targets was strictly based on the clustering results of the aforementioned phylogenetic tree. First, as indicated by the phylogenetic analysis, the winter sequence Win-parvo-Snow Leopard-2 clustered within the genus *Chaphamaparvovirus*. Accordingly, we aligned its NS1 protein against known representative strains within this genus. The SDT matrix (Figure 5A) revealed that the row and column corresponding to this sequence exhibited uniformly low-homology values, with its highest amino acid identity to any known *chaphamaparvovirus* in public databases reaching only 47.7%.

**FIGURE 5:**
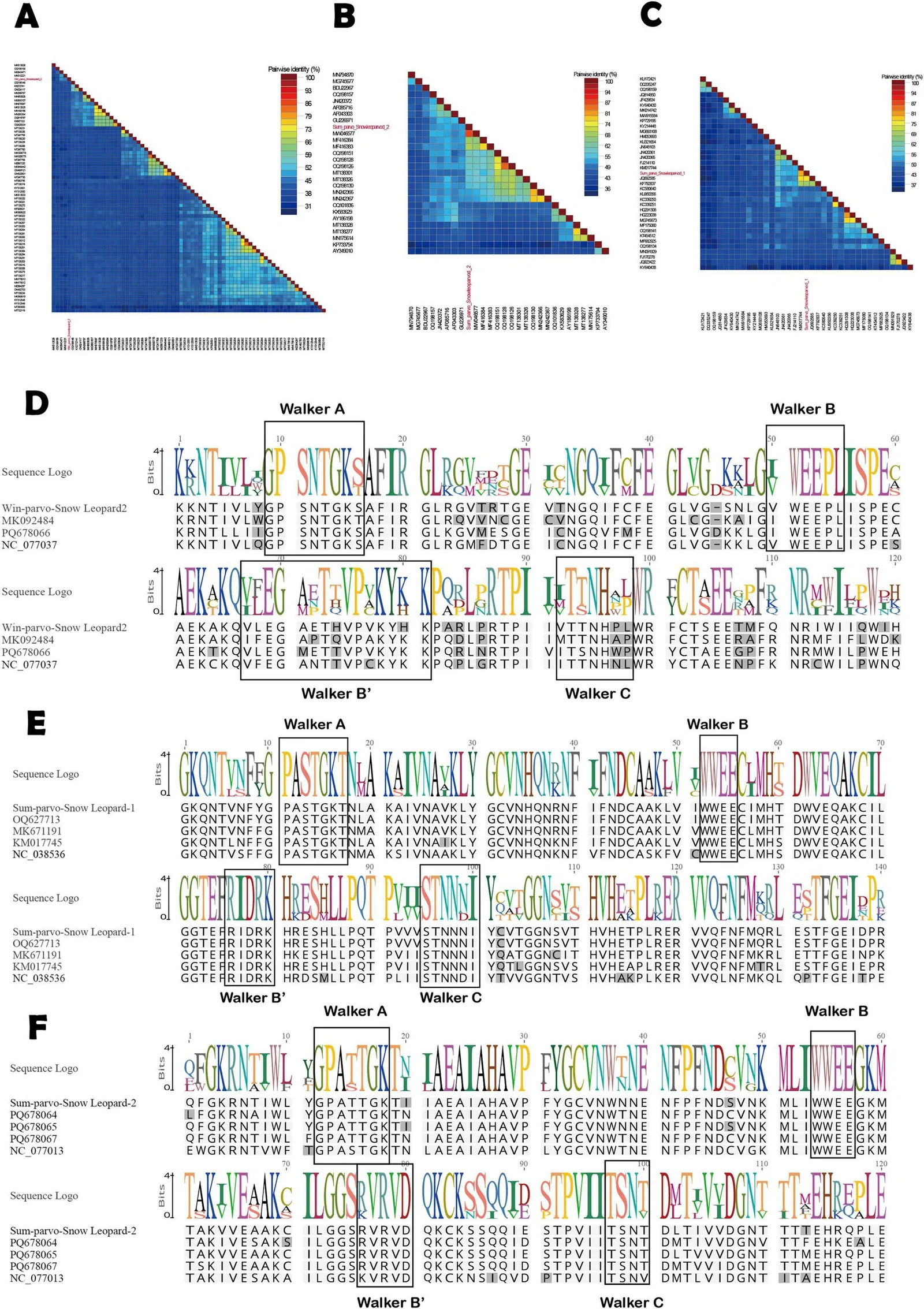
Comparative genome-wide and protein sequence identity analyses of the candidate novel parvoviruses. **(A–C)** Heatmaps showing pairwise identities between the three snow leopard parvovirus genomes and representative strains of *chaphamaparvovirus*, *bocaparvovirus*, and *dependoparvovirus*, respectively. **(D–F)** Sequence logos and NS1 amino acid alignments showing the conserved Walker A, Walker B, Walker B’, and Walker C motifs of Win-parvo-Snow Leopard-2, Sum-parvo-Snow Leopard-1, and Sum-parvo-Snow Leopard-2, respectively.

Next, an in-depth analysis was conducted for the two summer parvoviruses based on their genus-level affiliations in the phylogenetic tree. Since Sum-parvo-Snow Leopard-1 clustered within the genus *Bocaparvovirus*, we compared its NS1 sequence with those of known bocaparvoviruses. Matrix analysis demonstrated that the highest amino acid identity between this virus and known reference strains within the genus (including phylogenetically proximate feline-derived bocaviruses) was 80.5% (Figure 5B). Similarly, Sum-parvo-Snow Leopard-2 clustered with the genus *Dependoparvovirus* and formed a basal branch. SDT analysis further corroborated its extreme sequence divergence, showing a maximum identity of 53.4% with known *dependoparvoviruses* (Figure 5C).

According to the species demarcation criteria established by the Parvoviridae Study Group of the International Committee on Taxonomy of Viruses (ICTV), the threshold for amino acid sequence identity of the NS1 protein between different species within the same genus should be < 85% (Pénzes et al., 2020). Although the three snow leopard-derived parvoviruses identified in this study clustered into their respective genera on the phylogenetic tree, their NS1 amino acid identities all fell below the 85% interspecies demarcation threshold. Combining phylogeny and sequence identity evidence, the results support Win-parvo-Snow Leopard-2, Sum-parvo-Snow Leopard-1, and Sum-parvo-Snow Leopard-2 as candidate novel viral species within the genera *Chaphamaparvovirus*, *Bocaparvovirus*, and *Dependoparvovirus*, respectively.

To further evaluate the sequence annotation and intact coding capacity of these three divergent parvoviruses, we performed multiple sequence alignment analysis of the superfamily 3 (SF3) helicase core domains of their NS1 proteins (Figures 5D–F). The results revealed that, despite exhibiting extremely high divergence at the full-length amino acid level, all three parvoviruses retained the core catalytic components essential for DNA replication and unwinding in parvoviruses. Specifically, the amino acid compositions of the Walker A, Walker B, Walker B’, and Walker C motifs in the two summer parvoviruses assigned to the genera Bocaparvovirus and *Dependoparvovirus* (Sum-parvo-Snow Leopard-1 and Sum-parvo-Snow Leopard-2, respectively) were almost identical to those of reference strains within the same genera. Concurrently, the winter parvovirus assigned to the genus *Chaphamaparvovirus* (Win-parvo-Snow Leopard-2) also preserved the typical conserved motif profile of its genus.

### A putative novel species and a divergent strain within the family Picornaviridae

In this study, we assembled two picornavirus genomes from the libraries and constructed a phylogenetic tree based on RNA-dependent RNA polymerase (RdRp) (Figure 6A). Phylogenetic analysis revealed that both February snow leopard-derived picornaviruses were placed within the subfamily Kodimesavirinae; however, they underwent substantial independent divergence and were assigned to two different genera within this subfamily. First, win-picorna-snow leopard-2 was assigned to the genus *Sakobuvirus*. Topologically, this picornavirus formed a tight evolutionary cluster with multiple sakobuvirus strains from domestic cats (*Felis catus*) including the Portuguese strain NC_022802 and the Italian strain OQ556110, supported by high bootstrap confidence at the base (bootstrap support = 100%). Second, the other picornavirus, win-picorna-snow leopard-1, was assigned to the genus *Kobuvirus*. On the phylogenetic tree, this virus clustered within a large clade comprising kobuviruses from diverse mammals (particularly carnivores). It exhibited close phylogenetic associations with strains from the masked palm civet (GenBank no. OM451184), canines (GenBank no. AB861494 and JN387133), and the classical Aichivirus A1 (GenBank no. NC_074775), sharing a common ancestor with another kobuvirus strain (GenBank no. MK671314) isolated from a diarrheic domestic cat in the same tree.

**FIGURE 6:**
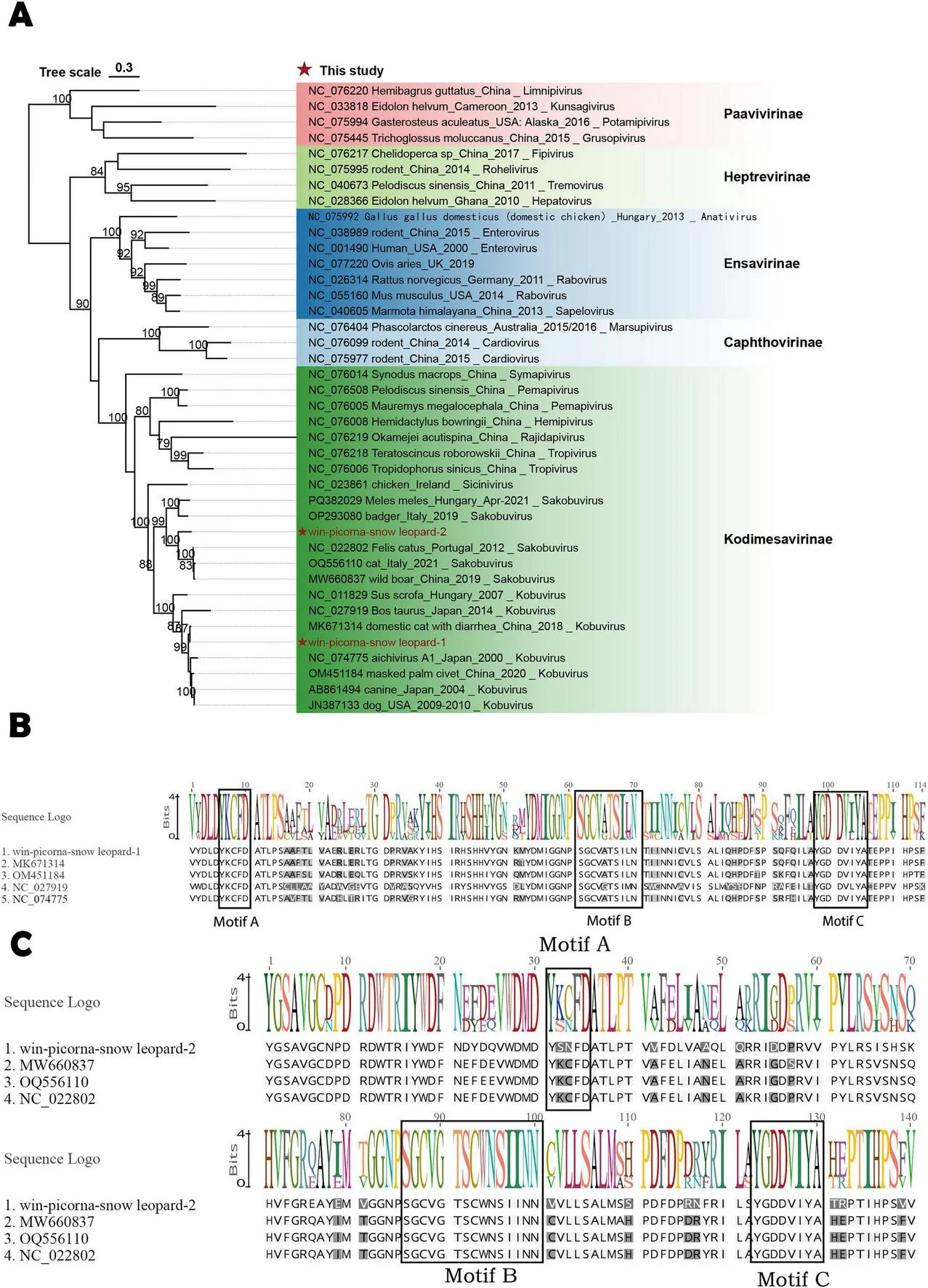
Phylogenetic and genomic characterization of picornaviruses identified in snow leopard feces. **(A)** Maximum-likelihood phylogenetic tree constructed from RdRp amino acid sequences using IQ-TREE 2. Node values represent support from 1,000 standard bootstrap replicates. The two genomes identified in this study are highlighted with red stars and cluster within the genera *Kobuvirus* and *Sakobuvirus* of the subfamily Kodimesavirinae. **(B,C)** Sequence logos and RdRp amino acid alignments showing conserved catalytic motifs A–C in win-picorna-snow leopard-1 and win-picorna-snow leopard-2.

To evaluate the taxonomic status of these two winter picornaviruses, we further conducted homology analyses of their key functional proteins. For the sequence assigned to sakobuvirus (win-picorna-snow leopard-2), we aligned the amino acid sequences of its P1 (capsid protein) and RdRp against the official ICTV reference strains of this genus (GenBank no. KF387721/NC_022802) and other highly homologous sequences from public databases. The results showed that its amino acid identities with the reference strains for P1 and RdRp were only 77.26% and 77.20%, respectively. According to the taxonomic criteria of the International Committee on Taxonomy of Viruses (ICTV)—which stipulate that amino acid identities for both P1 and RdRp between different species within the same genus must typically be < 80%–this sequence divergence supports win-picorna-snow leopard-2 as a candidate novel species within the genus *Sakobuvirus* (Zell et al., 2017). In contrast, the sequence assigned to *Kobuvirus* (win-picorna-snow leopard-1) shared an RdRp amino acid identity of > 80% with the known reference strain (GenBank no. KF831027). Therefore, based on the same classification thresholds, this sequence is best interpreted as a divergent strain of a known species detected in a snow leopard fecal sample.

Based on the phylogenetic and sequence comparisons, the two picornaviruses were interpreted as a candidate novel species and a divergent strain, respectively. To evaluate conservation of their annotated polymerase regions, we conducted a conserved motif analysis on the core catalytic domains of their continuous RdRp sequences (Figures 6B,C). Multiple sequence alignments and Sequence Logo results demonstrated that, despite a highly variable genetic background, both snow leopard-derived viruses retained the classical catalytic motifs essential for nucleic acid chain elongation in picornaviruses.

### Identification of four highly divergent Genomoviridae genomes

In this study, we assembled four full-length genomes of genomovirus from the libraries and constructed a phylogenetic tree based on the amino acid sequences of the replication-associated protein (Rep) (Figure 7). The phylogenetic topology revealed that the four snow leopard-derived genomoviruses obtained in this study not only failed to form a host-specific independent clade but rather exhibited substantial phylogenetic divergence. They fell into four distinct viral genera and clustered with reference strains of environmental, plant, or non-feline origins. Specifically, the winter genomovirus Win-Gen-Snow Leopard-1 was located at the most basal branch of the genus *Gemycircularvirus*, sharing the closest evolutionary affinity with reference strains derived from water buffalo (*Bubalus bubalis*) and goldenrod (*Solidago* sp., plant) (bootstrap support = 100%). Another winter genomovirus, Win-Gen-Snow Leopard-2, was assigned to the genus *Gemykrogvirus*, forming a highly robust cluster on the evolutionary tree with viruses isolated from airborne particulate matter, sewage, and bats (*Rhinolophus*) (bootstrap support = 100%). Among the summer genomoviruses, the virus Sum-Gen-Snow Leopard-2 was placed into the genus *Gemygorvirus*, clustering within the same major clade as strains broadly derived from birds (e.g., domestic chicken Gallus, rustic bunting *Emberiza rustica*), humans, and environmental samples (node bootstrap support = 100%). Meanwhile, the other virus Sum-Gen-Snow Leopard-1 fell into the genus *Gemykibivirus*, with its closest reference strains originating from the desert tortoise (*Gopherus agassizii*), black cutworm (*Agrotis ipsilon*, a moth insect), and finches, together constituting a monophyletic group with high confidence (bootstrap support = 100%).

**FIGURE 7:**
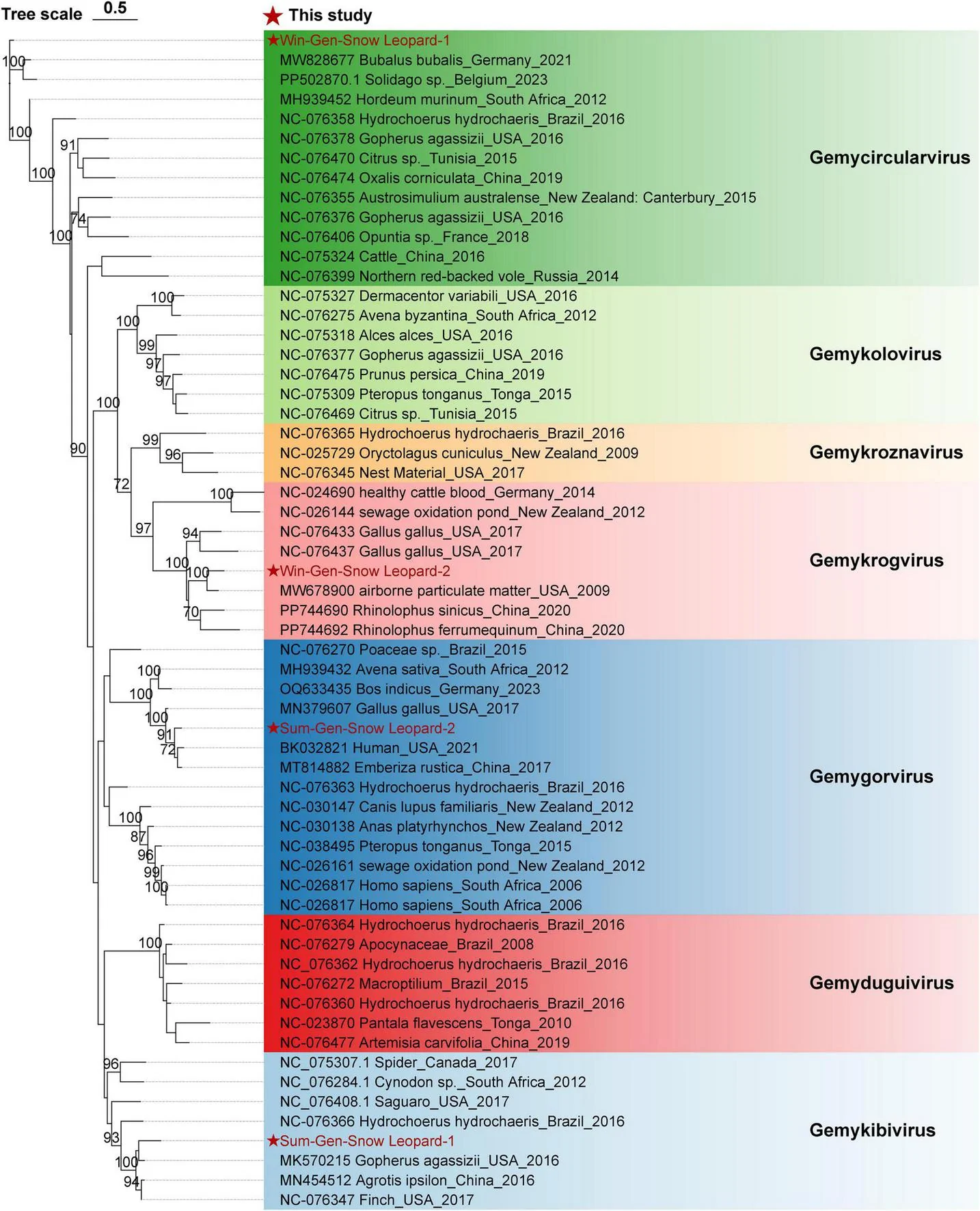
Phylogeny of genomoviruses identified in snow leopard feces. The maximum-likelihood tree was constructed from Rep amino acid sequences using IQ-TREE 2. Node values represent support from 1,000 standard bootstrap replicates. The four genomes identified in this study are highlighted with red stars and fall within the genera *Gemycircularvirus*, *Gemykrogvirus*, *Gemygorvirus*, and *Gemykibivirus*.

To further evaluate the taxonomic status of these four genomoviruses at the species level, we utilized the SDT software to calculate their sequence pairwise identity matrices against known representative reference strains within their respective genera. First, as indicated by the phylogenetic analysis, the winter sequence Win-Gen-Snow Leopard-1 clustered within the genus *Gemycircularvirus*. Based on this, we aligned its sequence against known representative strains within this genus. The SDT matrix (Figure 8A) demonstrated that the row and column corresponding to this sequence exhibited uniformly dark blue, low-homology characteristics, with its highest identity to any known gemycircularvirus in public databases reaching only 71.3%. Next, for the other winter sequence, Win-Gen-Snow Leopard-2, we similarly conducted an analysis based on its genus-level affiliation on the evolutionary tree. Matrix analysis (Figure 8B) revealed that the highest identity between this sequence and known reference strains within the genus *Gemykrogvirus* was 61.2%. Similarly, the two summer sequences, Sum-Gen-Snow Leopard-2 and Sum-Gen-Snow Leopard-1, clustered with the genera *Gemygorvirus* and *Gemykibivirus* on the phylogenetic tree, respectively. SDT analysis (Figures 8C,D) further supported their sequence divergence, showing that the highest identities of these two sequences with their respective known reference strains were 60.2% and 68.4%, respectively.

**FIGURE 8:**
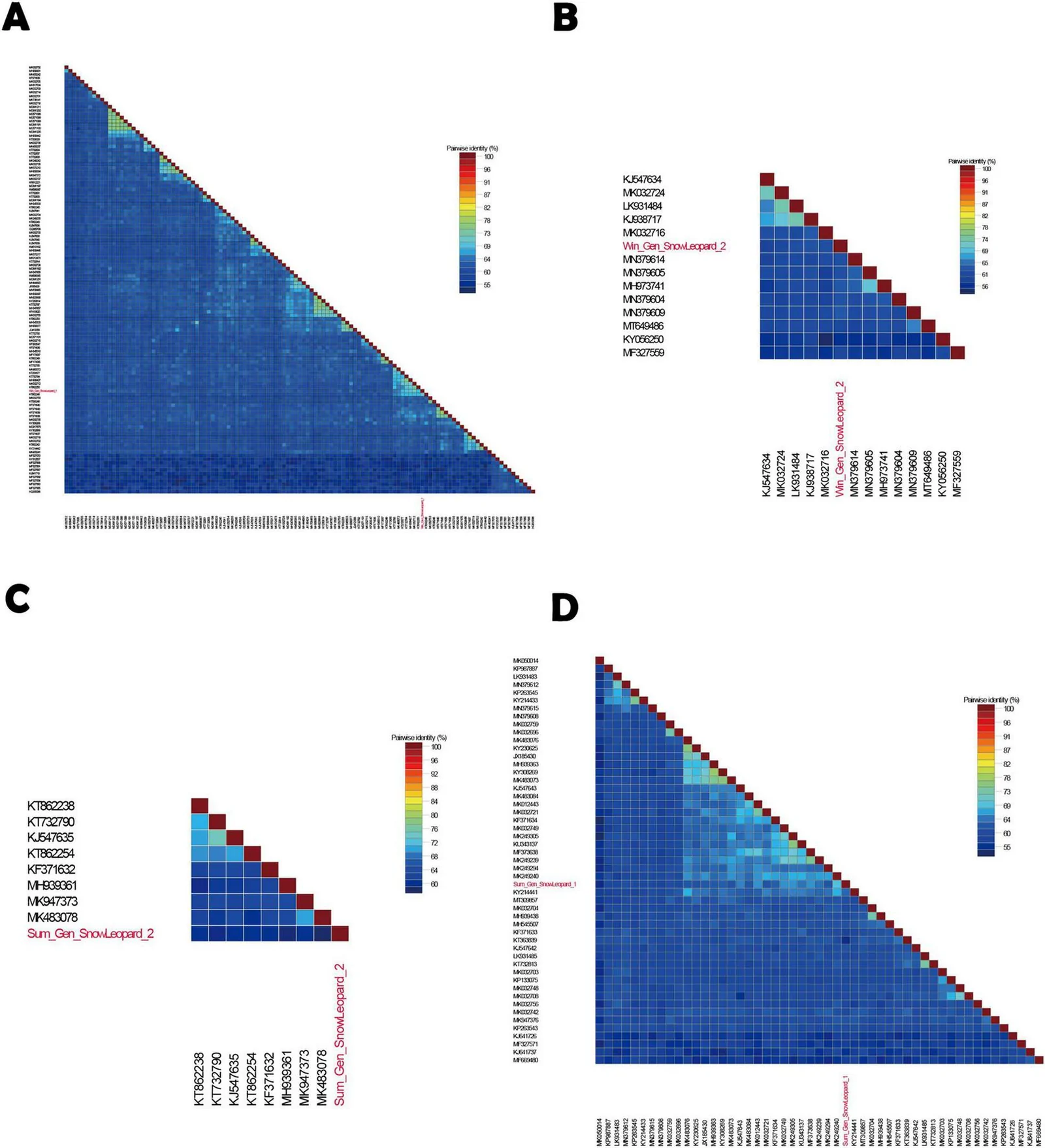
Genomic characterization of genomoviruses identified in snow leopard feces. **(A–D)** Heatmaps showing genome-wide pairwise nucleotide identity between the snow leopard genomovirus genomes and representative strains from their assigned genera: **(A)** Win-Gen-Snow Leopard-1 and *Gemycircularvirus*; **(B)** Win-Gen-Snow Leopard-2 and *Gemykrogvirus*; **(C)** Sum-Gen-Snow Leopard-2 and *Gemygorvirus*; and **(D**) Sum-Gen-Snow Leopard-1 and *Gemykibivirus*.

According to the species demarcation criteria established by the International Committee on Taxonomy of Viruses (ICTV) for the family Genomoviridae, the genome-wide sequence identity threshold for different species within the same genus should be < 78% (Varsani and Krupovic, 2017). Although the four snow leopard-derived genomovirus sequences identified in this study clustered into their corresponding genera on the phylogenetic tree, their sequence identities all fell far below the 78% interspecies demarcation line. Synthesizing the dual evidence of phylogeny and sequence identity, the results support Win-Gen-Snow Leopard-1, Win-Gen-Snow Leopard-2, Sum-Gen-Snow Leopard-2, and Sum-Gen-Snow Leopard-1 as candidate novel viral species within the genera *Gemycircularvirus*, *Gemykrogvirus*, *Gemygorvirus*, and *Gemykibivirus*, respectively.

### Identification of a candidate novel *Porprismacovirus* with a putative recombination signal

In this study, we identified a smacovirus, designated Sum-sma-SnowLeopard-1, in one summer sample and constructed a phylogenetic tree based on its Rep protein (Figure 9A). The phylogenetic topology placed this sequence within the major clade of the genus *Porprismacovirus*, with 100% bootstrap support at the corresponding basal node. Within this genus, Sum-sma-SnowLeopard-1 did not cluster closely with the available sequences derived from other wild felids, such as those detected in the Tanzanian lion (*Panthera leo*) or the bobcat (*Lynx rufus*). Instead, it formed a relatively distinct branch and showed a closer phylogenetic relationship with reference sequences derived from domestic chickens (*Gallus gallus*; GenBank no. MN379618) and domestic pigs (*Sus scrofa domesticus*; e.g., GenBank no. NC_076238), together forming a subclade with 94% bootstrap support.

**FIGURE 9:**
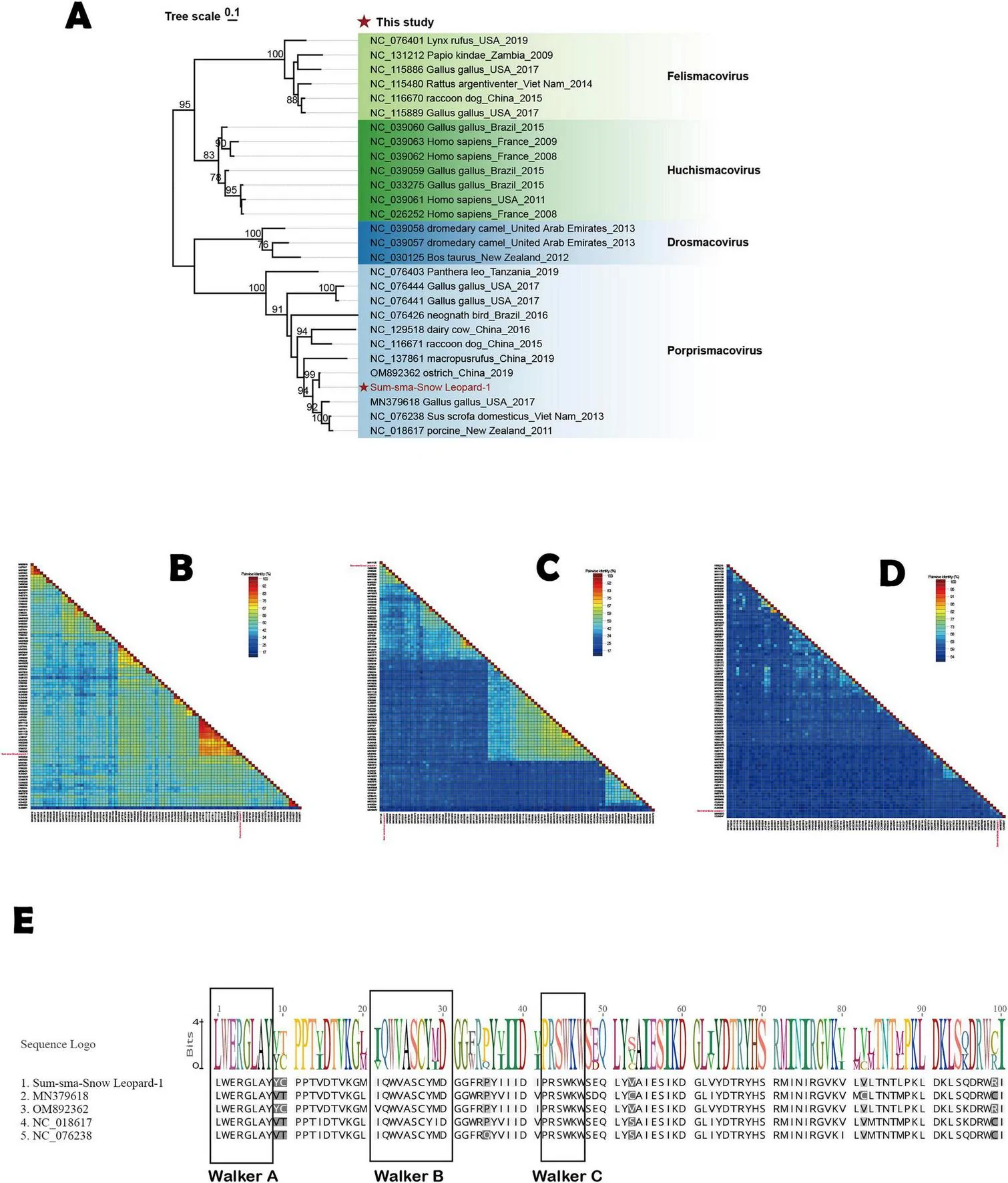
Phylogenetic and genomic characterization of Sum-sma-SnowLeopard-1. **(A)** Maximum-likelihood phylogenetic tree constructed from Rep amino acid sequences using IQ-TREE 2. Node values represent support from 1,000 standard bootstrap replicates. **(B–D)** Heatmaps showing pairwise identities for the Cap protein, Rep protein, and complete genome between Sum-sma-SnowLeopard-1 and representative *Porprismacovirus* sequences. **(E)** Sequence logo and Rep amino acid alignment showing the conserved Walker A–C motifs.

To further evaluate the taxonomic status of this smacovirus, we performed SDT pairwise identity comparisons of its encoded proteins and complete genome with the corresponding sequences of previously reported smacoviruses. The Cap protein of Sum-sma-SnowLeopard-1 exhibited substantial sequence divergence, with a maximum amino acid identity of 59.701% to the reference sequences available in public databases (Figure 9B). In contrast, its Rep protein was considerably more conserved, reaching a maximum amino acid identity of 96.701% (Figure 9C). Whole-genome comparisons showed a maximum nucleotide identity of 63.601% with the representative smacovirus sequences included in the analysis (Figure 9D). According to the species-demarcation criteria proposed for the family Smacoviridae, an 80% genome-wide pairwise nucleotide identity threshold is generally applied to distinguish species within a genus (Varsani and Krupovic, 2018). Because the maximum whole-genome identity of Sum-sma-SnowLeopard-1 was substantially below this threshold, the sequence is consistent with a candidate novel species within the genus *Porprismacovirus*. The contrasting levels of conservation between the Rep and Cap proteins were also compatible with a possible mosaic evolutionary history and therefore prompted dedicated recombination analyses.

RDP4 identified a putative recombinant interval spanning alignment positions 1598–3018, corresponding to nucleotide positions 1010–2121 of the 2,420-nt Sum-sma-SnowLeopard-1 genome (Figure 10A). MT457882 and OM892362 were identified as the candidate major and minor parental sequences, respectively. The event received significant support from seven independent algorithms—RDP, GENECONV, BootScan, MaxChi, Chimaera, SiScan, and 3Seq—with individual *P* values ranging from 1.11 × 10^–46 to 7.88 × 10^–12 (Figure 10F), whereas PhylPro and LARD did not reach statistical significance. The BootScan profile showed a corresponding shift in phylogenetic affinity across the inferred recombinant interval (Figure 10B), and the SiScan component-score profile displayed a coincident signal spanning the same region (Figure 10C).

**FIGURE 10:**
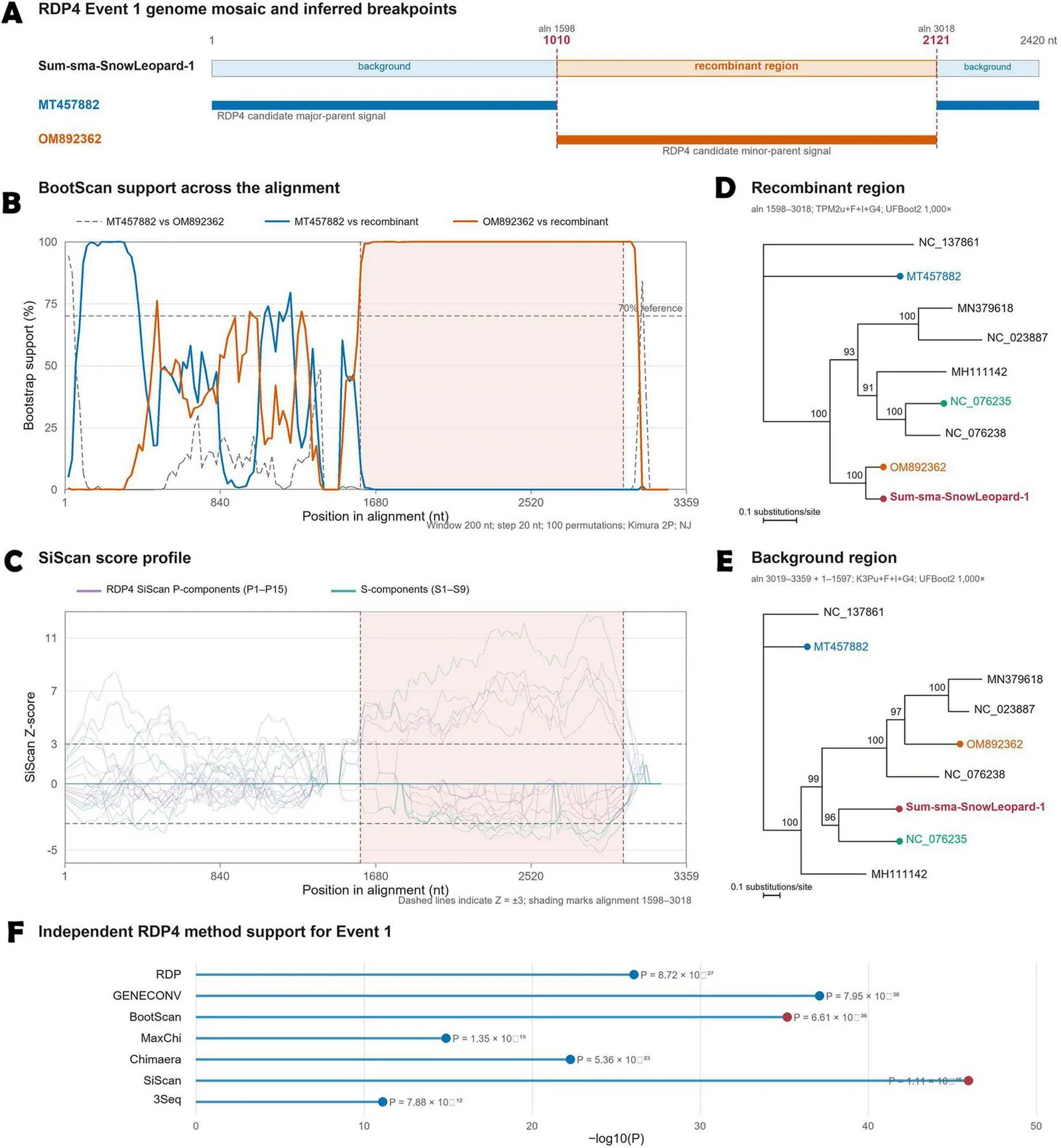
Bioinformatic evidence for a putative recombination event in Sum-sma-SnowLeopard-1. **(A)** RDP4-inferred recombinant region (genome positions 1010–2121; alignment positions 1598–3018), with MT457882 and OM892362 as the candidate major and minor parental sequences, respectively. **(B)** BootScan profile across the inferred region (window, 200 nt; step, 20 nt; 100 replicates). Shading and vertical dashed lines indicate the recombinant region and breakpoints. **(C)** SiScan profile, with horizontal dashed lines indicating Z-scores of ± 3. **(D,E)** Maximum-likelihood trees of the recombinant and background regions, respectively. Sum-sma-SnowLeopard-1 clustered with OM892362 in the recombinant-region tree (100% support) and with NC_076235 in the background-region tree (96% support). **(F)** Event-specific *P* values obtained using seven RDP4 detection methods.

Region-specific phylogenetic analyses further supported this change in evolutionary affinity. In the tree reconstructed from the recombinant region, Sum-sma-SnowLeopard-1 clustered with OM892362 with 100% ultrafast bootstrap support (Figure 10D). In contrast, the tree based on the remaining background region placed Sum-sma-SnowLeopard-1 with NC_076235 with 96% ultrafast bootstrap support (Figure 10E). The placement of the sequence with NC_076235 rather than MT457882 in the background-region tree is consistent with the candidate-parent assignment representing the closest recombination signal identified by RDP4 among the sequences analyzed, rather than a direct ancestral relationship. In addition, read remapping showed continuous coverage and concordant read support across both inferred breakpoints, reducing the likelihood that the observed pattern resulted from an unsupported contig join. Collectively, the concordant breakpoint signals, statistically significant support from multiple recombination-detection algorithms, region-specific phylogenetic incongruence, and read-remapping evidence support a putative mosaic genome structure for Sum-sma-SnowLeopard-1.

To further characterize the conserved features of the Rep protein, we performed multiple-sequence alignment and motif analysis of the amino acid region encompassing the superfamily 3 helicase catalytic domains (Figure 9E). Despite the substantial divergence observed at the whole-genome and Cap-protein levels, the Rep protein retained the three conserved catalytic motifs associated with rolling-circle replication in CRESS DNA viruses: Walker A (LWERGLAY), Walker B (IQWVASCYMD), and the Walker C core region (PRSW). At these conserved sites, Sum-sma-SnowLeopard-1 showed high amino acid similarity to avian-derived reference sequences, including MN379618 and OM892362, and porcine-derived sequences, including NC_076238 and NC_018617.

## Discussion

In recent years, next-generation sequencing (NGS) technologies have been widely applied in virology, enabling viral metagenomic sequencing to identify various known and previously undescribed viruses and thereby improving our understanding of animal virome diversity, community structure, and evolutionary relationships (Zhang et al., 2018; French and Holmes, 2020). Based on this advanced technical approach, characterizing the gut virome diversity of wildlife is important for investigating viral evolutionary trajectories and expanding knowledge of wildlife-associated viruses. However, for rare apex predators inhabiting high-altitude ecosystems such as the Qinghai-Tibet Plateau, the composition of their fecal viromes remains largely unknown. Through metagenomic analysis, this study characterized the diverse viral communities detected in fecal samples from captive snow leopards at the Qinghai Wildlife Park and identified 22 complete viral genomes representing multiple candidate novel species or divergent strains across six viral families. Within these communities, circular Rep-encoding single-stranded DNA (CRESS DNA) viruses (e.g., Anelloviridae, Circoviridae, Smacoviridae, and Genomoviridae) and Parvoviridae accounted for a large proportion of the classified viral reads. Notably, these predominant viruses were single-stranded DNA and non-enveloped viruses. It is conceivable that these relatively simple genome and virion characteristics may contribute to environmental stability and partly explain their frequent detection in fecal and environmental samples. These newly identified viruses expand the available genomic information for CRESS DNA viruses and parvoviruses detected in felid feces and warrant further investigation of their host associations and evolutionary relationships.

Given that the gut microecology of captive wildlife is chronically exposed to artificial dietary interventions and restricted spatial mobility, accurately distinguishing true endogenous viral infections from transient environmental or dietary passage is crucial for deciphering virome ecology. The candidate novel bocaparvovirus (Sum-parvo-Snow Leopard-1) identified in our summer samples exhibited phylogenetic features compatible with a felid-associated lineage. The family Parvoviridae comprises non-enveloped, single-stranded linear DNA viruses with icosahedral symmetry. Within the subfamily Parvovirinae, the genus *Bocaparvovirus* is distinguished by its unique three open reading frame (ORF) structure and high frequencies of genetic recombination (Cotmore et al., 2014). Bocaparvoviruses have been associated with fecal-oral transmission and cross-species infections, causing severe respiratory and gastrointestinal diseases in juvenile hosts or establishing asymptomatic infections in adults. Phylogenetically, this candidate novel bocaparvovirus showed strongly supported basal clustering with reference strains recently identified in other wild or stray felines. This supports the hypothesis that related bocaparvoviruses circulate among felids and motivates longitudinal investigation of persistence and host association in snow leopards. In contrast to the potentially pathogenic characteristics of bocaparvoviruses, the family Anelloviridae is generally considered a highly divergent commensal viral community lacking definitive pathogenicity. Anelloviruses possess the smallest and simplest circular single-stranded negative-sense DNA genomes among known animal-infecting viruses, exhibiting broad epidemiological prevalence and a long, complex co-evolutionary history with mammalian hosts (Kaczorowska and van der Hoek, 2020). In this study, we identified 9 divergent Anellovirus genomes representing multiple candidate novel taxa across both summer and winter samples. This discovery expands the genomic landscape of anelloviruses in wild felines and shows that divergent anellovirus sequences can be detected in snow leopard feces at both sampling times, providing candidates for longitudinal studies of host association and persistence.

In addition to viruses exhibiting host-associated phylogenetic patterns, certain taxa detected in fecal metagenomes may reflect the host’s transient exposure to the environment and diet. The family Circoviridae belongs to the CRESS DNA viruses and exhibits a broad host range across vertebrates and invertebrates (Rosario et al., 2017). Based on the phylogenetic topologies in this study, the July snow leopard-derived circovirus sequences formed a monophyletic clade with avian-derived viruses, whereas the February sequence clustered with viruses reported from mammalian carnivores, including dogs, wolves, and foxes. Combined with the broad host distribution of circoviruses, this sampling-period-associated pattern is consistent with dietary passage as one possible explanation and provides a basis for future paired analyses of fecal, feed, and environmental samples. Similarly, the four candidate novel species within the family Genomoviridae showed phylogenetic relationships with sequences derived from diverse ecological sources. The reported host range of genomoviruses encompasses fungi, plants, insects, and vertebrates, and these viruses have also been detected in environmental samples (Varsani and Krupovic, 2017). The four snow leopard-derived sequences did not form a feline-exclusive evolutionary branch but clustered with reference strains originating from sewage, airborne particulate matter, plants, and non-feline animals, including bats and birds. Such broad phylogenetic relationships are compatible with several possible environmental or dietary origins and provide useful clues for subsequent comparative source-tracing studies. Collectively, these findings highlight potential relationships between the fecal virome of captive snow leopards and their dietary and environmental exposures.

The human-animal interface in zoological parks not only increases multi-source environmental exposure but also may create opportunities for contact among viruses from different ecological sources. The novel porprismacovirus species identified within the family Smacoviridae showed a putative recombination signal. Strikingly, the replication-associated protein (Rep) of this snow leopard-derived strain exhibited extreme conservation (up to 96.7% identity) with avian- and porcine-derived reference strains, yet its capsid protein (Cap) and overall genomic sequence displayed extreme divergence. Such Rep-Cap discordance is compatible with recombination and provides a useful framework for evaluating the contributions of recombination, differential evolutionary rates, and region-specific selective pressures. RDP4, SimPlot, region-specific phylogenies, and read remapping provided additional support for a mosaic genome structure. The background tree instead grouped the sequence with NC_076235 rather than the RDP4-proposed major parent, highlighting a potentially broader parental sequence space for future analysis. Retention of the Walker A/B/C motifs and continuous read coverage support an intact assembly and a conserved replication-associated domain architecture (Varsani and Krupovic, 2018).

Beyond the diversity and recombination of DNA viruses, the potential cross-species transmission of enteric RNA viruses is also relevant to captive-wildlife conservation. The family Picornaviridae comprises non-enveloped, positive-sense single-stranded RNA viruses, some of which are associated with gastrointestinal or respiratory disease in mammals (Reuter et al., 2011). In this study, we assembled a candidate novel sakobuvirus species and a divergent kobuvirus strain from the February samples. In the phylogenetic analysis, these viruses clustered with reference picornaviruses previously reported from domestic cats, dogs, pigs, and other mammals. These relationships raise testable hypotheses concerning shared sources or transmission and identify epidemiologically linked animals, feed, water, and environmental samples as priorities for future investigation. Multiple-sequence alignments showed conservation of the RdRp catalytic motifs in both genomes. These observations support continued surveillance at multi-species interfaces and provide a basis for targeted evaluation of possible spillover into snow leopards.

This study systematically delineates the complex composition of the cryptic gut virome in captive snow leopards at high altitudes and also identifies several considerations that can guide subsequent work. Given the inherent challenges of sampling rare wildlife, the seven samples from a single zoo provide a focused baseline for future comparisons with larger and free-ranging snow leopard populations. The 0.45 μm membrane filtration used for viral enrichment enhanced sequencing depth for the targeted fraction and suggests that complementary multi-pore-size enrichment could broaden future recovery of nucleocytoplasmic large DNA viruses (NCLDVs) (Conceição-Neto et al., 2018). Building on this baseline, expanded longitudinal sampling across spatial and temporal dimensions, together with quantitative assays, strand-specific detection where relevant, viral isolation, serology, and host-response data, will enable more comprehensive evaluation of the epidemiological significance and pathogenic potential of these viral variants at the multi-species interface.

## Conclusion

This study recovered 22 complete viral genomes from seven captive snow leopard fecal samples and documented substantial diversity across six viral families. Sequence comparisons supported multiple candidate novel taxa, and complementary analyses supported a putative mosaic genome structure in Sum-sma-SnowLeopard-1, prioritizing this lineage for broader parental-sequence sampling. These data establish a genomic baseline for a localized captive population and provide a basis for larger, individually matched and biologically validated studies.

## Data Availability

The raw sequencing data generated in this study have been deposited in the NCBI Sequence Read Archive (SRA) under accession numbers SRR40370603-SRR40370609. The 22 complete viral genome sequences generated in this study have been deposited in GenBank under accession numbers PZ516756-PZ516777. Additional genomic characteristics and ORF annotations are provided in Supplementary Table 1.

## Footnotes

1
https://www.ncbi.nlm.nih.gov/Structure/cdd/wrpsb.cgi

2
https://www.chiplot.online/

## Supplementary material

SUPPLEMENTARY TABLE 1 Genomic characteristics and ORF annotations of the virus sequences identified in this study.
